# Supplementary material for: Variability in X-ray induced effects in [Rh(COD)Cl]2 with changing experimental parameters†
Source: Phys Chem Chem Phys. Author manuscript; Available in PMC 2023 Jan 23. (PMC7614095; doi:10.1039/d2cp03928a)
Supplement: Supplemetary information [file EMS160050-supplement-Supplemetary_information.pdf]

Electronic Supplementary Material (ESI) for Physical Chemistry Chemical Physics.
This journal is © the Owner Societies 2022

Supplementary Information
Variability in X-ray induced effects in $[Rh(COD)Cl]_2$ with changing experimental parameters

Nathalie K. Fernando,[a*] Hanna Boström,[b] Claire A. Murray,[c] Robin L. Owen,[c] Amber L. Thompson,[d] Joshua L. Dickerson,[e] Elspeth F. Garman,[f] Andrew B. Cairns,[g] and Anna Regoutz[a*]

[a] *Department of Chemistry, University College London, 20 Gordon Street, London, WC1H 0AJ, UK.*

[b] *Max Planck Institute for Solid State Research, Heisenbergstraße 1, 70569 Stuttgart, Germany.*

[c] *Diamond Light Source Ltd, Diamond House, Harwell Science and Innovation Campus, Didcot, Oxfordshire, OX11 0DE, UK.*

[d] *Chemical Crystallography, Chemistry Research Laboratory, University of Oxford, South Parks Road, Oxford OX1 3QR, UK.*

[e] *MRC Laboratory of Molecular Biology, Francis Crick Avenue, Cambridge Biomedical Campus, Cambridge CB2 0QH, UK.*

[f] *Department of Biochemistry, Dorothy Crowfoot Hodgkin Building, University of Oxford, South Parks Road, Oxford, OX1 3QU, UK.*

[g] *Department of Materials, Imperial College London, Royal School of Mines, Exhibition Road, SW7 2AZ, UK.*

1 Estimation of Dose

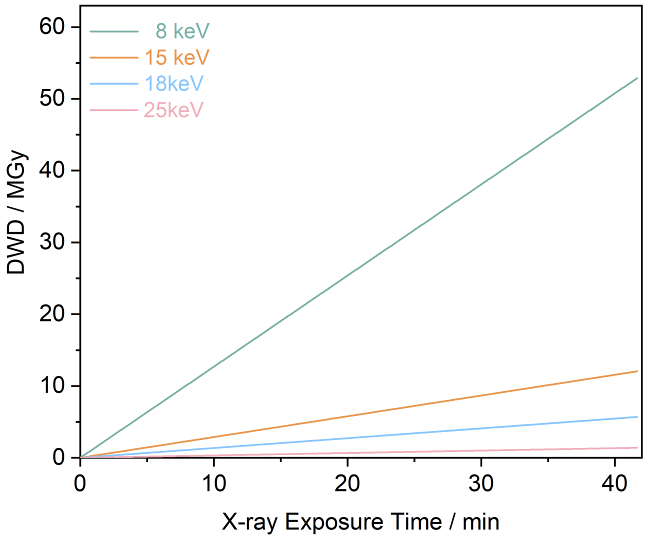

Figure S1: The absorbed diffraction-weighted dose (DWD) calculated using RADDOSE-3D, as a function of X-ray exposure time for $[Rh(COD)Cl]_2$, at photon energies $h\nu$ of 8, 15, 18, and 25 keV.

Table S1: The parameters included in the RADDOSE-3D input file for $[Rh(COD)Cl]_2$ used to estimate the doses for the varying photon energy setups in the powder XRD experiments at beamline I11 at Diamond Light Source, Didcot, UK.

RADDOSE parameter	8 keV	15 keV	18 keV	25 keV
Crystal type	cylinder	cylinder	cylinder	cylinder
Crystal Dimension* / μm	300 × 40000	300 × 40000	300 × 40000	300 × 40000
PixelsPerMicron	0.06	0.06	0.06	0.06
Container material type	mixture	mixture	mixture	mixture
Material mixture	pyrex	pyrex	pyrex	pyrex
Container thickness / μm	10	10	10	10
Container density / $g\,cm^{-3}$	2.23	2.23	2.23	2.23
Beam type	Gaussian	Gaussian	Gaussian	Gaussian
Photon flux / $ph\,s^{-1}$	5.7×10^{12}	2.4×10^{12}	1.4×10^{12}	2.2×10^{11}
FWHM / μm^2	2000 × 600	2000 × 600	2000 × 600	2000 × 600
Energy / keV	8	15	18	25
Collimation type	rectangular	rectangular	rectangular	rectangular
Collimation dimensions / μm^2	2500 × 800	2500 × 800	2500 × 800	2500 × 800

* diameter × length of capillary

2 PXRD Refinements

The small, periodic discontinuities observed in the data, particularly evident in the 8 keV and 15 keV integrated intensity (peak area) plots as a function of time and dose (Figures S3(a) and S3(d)) can be attributed to changes in beam current associated with the top-up mode of the synchrotron storage ring.

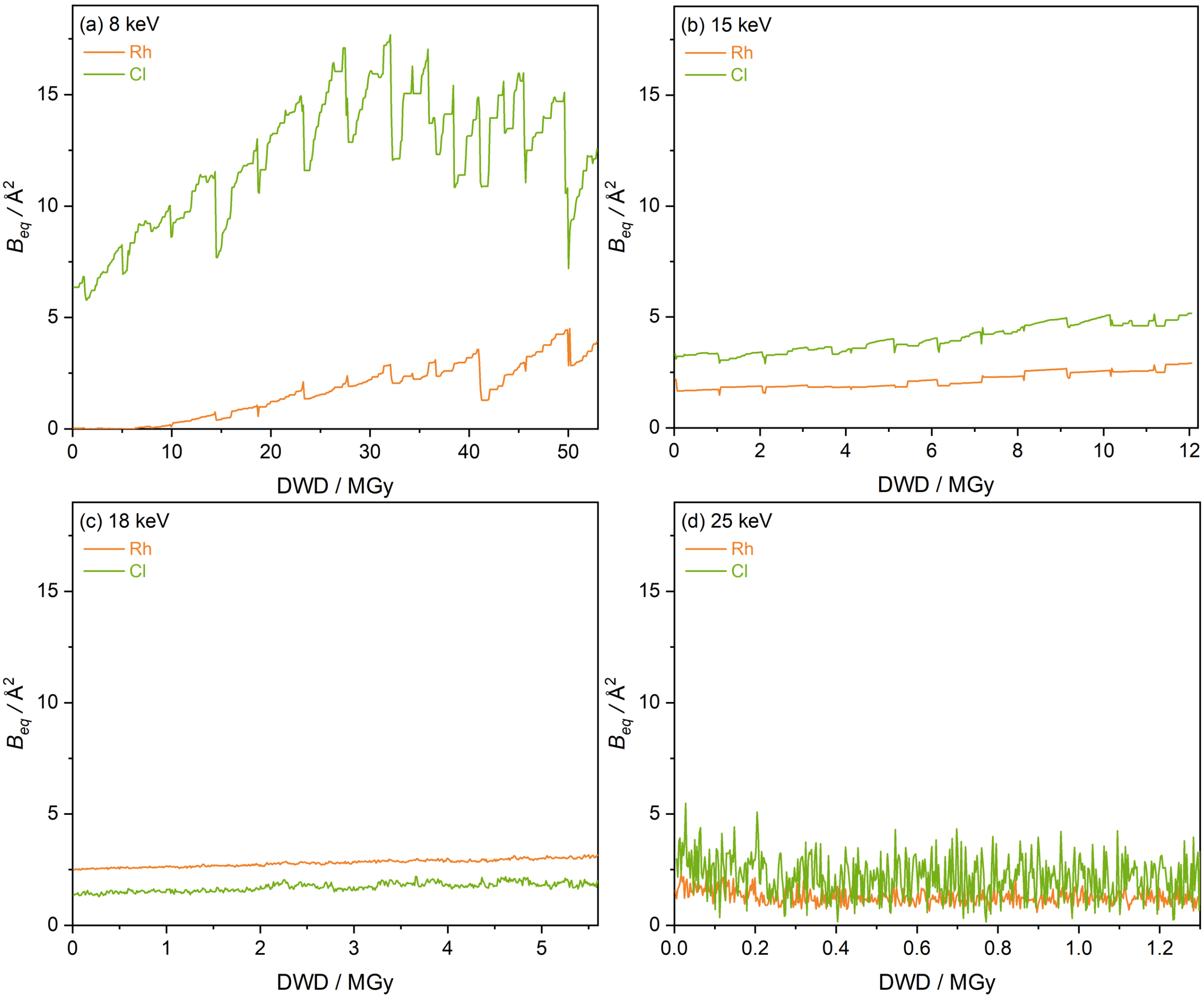

Figure S2: The refined values of the isotropic atomic displacement parameter B_{eq} for Rh and Cl at setups with a photon energy $h\nu$ of (a) 8 keV, (b) 15 keV, (c) 18 keV and (d) 25 keV as a function of diffraction-weighted dose (DWD). The carbon B_{eq} values are fixed to a value of 2.0 throughout all 500 datasets.

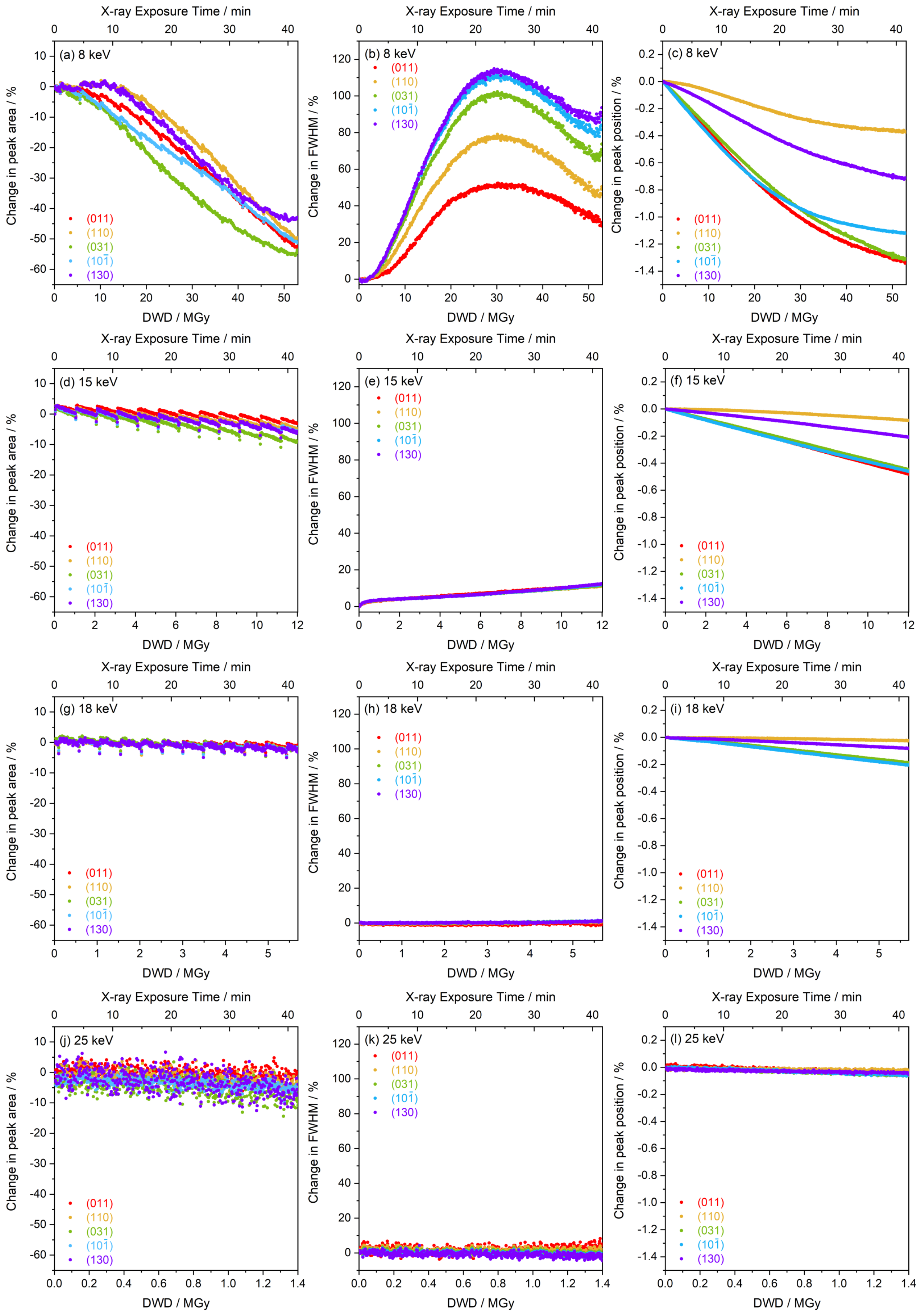

Figure S3: The percentage change in peak intensity, FWHM, and peak shift for $[Rh(COD)Cl]_2$ as a function of diffraction-weighted dose (DWD) and X-ray exposure time, obtained from Le Bail refinements, at setups with a photon energy $h\nu$ of (a) 8 keV, (b) 15 keV, (c) 18 keV and (d) 25 keV.

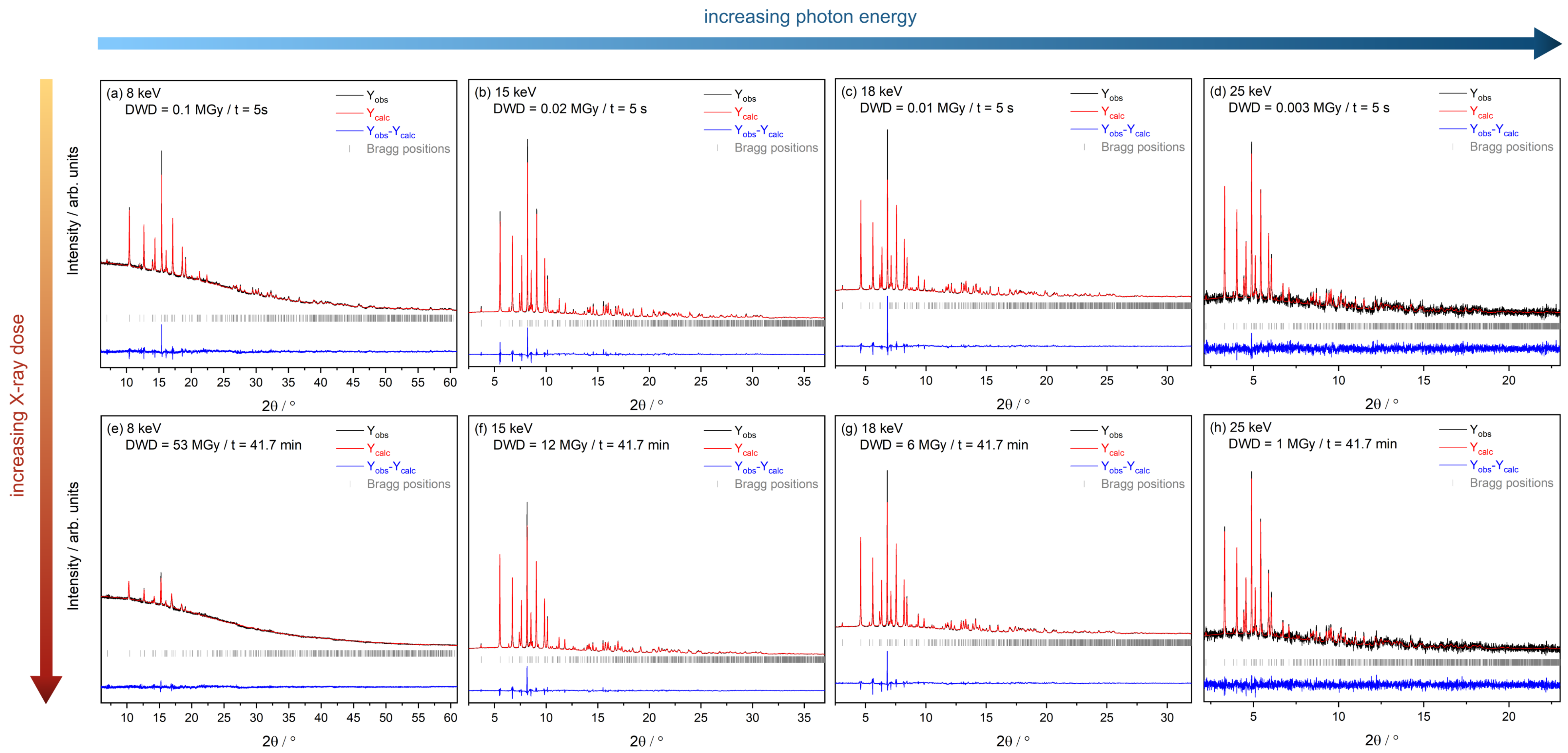

Figure S4: The Rietveld refinements of the minimum dose (top row) and maximum dose (bottom row) PXRD patterns of $[Rh(COD)Cl]_2$ at experimental setups with a photon energy $h\nu$ of (a) and (e) 8 keV, (b) and (f) 15 keV, (c) and (g) 18 keV, and (d) and (h) 25 keV.

3 Estimation of PXRD Resolution

In single crystal macromolecular X-ray diffraction, discussion of resolution is routine, particularly in investigations of X-ray damage. However, this is not the case in powder X-ray diffraction studies. Although there is no direct equivalent, in order to bridge the gap between the two techniques, a somewhat crude approximation of PXRD resolution is made here. This was achieved by extracting the average error on the raw intensities, σ, obtained during Rietveld refinements. The diffraction peak at the furthest 2θ point, which has an intensity above the 2σ threshold is considered to be the last resolvable peak,[1] and converted to the q 'resolution' value. Tabulated below are the estimated q values at each photon energy studied at the start ($t = 5\,\mathrm{s}$) and end ($t = 41.7\,\mathrm{min}$) of irradiation. It should be noted that due to the organic and monoclinic nature of the complex, individual peaks at high 2θ values could not be completely resolved. Therefore the errors in the d-spacing are defined by the half-width of these broad peaks, which takes into account the 2θ distribution of underlying peaks.

Table S2: Estimation of the 'resolution' of the diffraction experiments carried out in this work extracted from our X-ray diffraction data. The peak at the furthest 2θ point is shown for each start and end diffraction pattern at the four energies studied, which has an intensity above the 2σ threshold, i.e. the last resolvable peak. This is tabulated with the associated Miller indices *hkl*, d-spacing, *d*, the error on *d*, resolution, *q* with its propagated error Δq and the percentage change in *q* from the minimum dose (start) to maximum dose (end) structure.

$h\nu$ / keV	2θ / °	$h\ k\ l$	d / Å	Δd	q / Å^{-1}	Δq	change in q / %
8_{start}	60.1	1 15 2	1.55	0.01	4.06	0.03	−61.8
8_{end}	22.1	0 3 2	4.04	0.04	1.56	0.02	
15_{start}	48.1	7 6 0	1.014	0.004	6.20	0.02	−19.5
15_{end}	38.3	$2\ 13\ \bar{5}$	1.26	0.01	4.98	0.05	
18_{start}	35.5	$6\ 7\ \bar{2}$	1.126	0.004	5.58	0.02	−7.0
18_{end}	33.0	6 2 0	1.211	0.007	5.19	0.03	
25_{start}	14.9	$2\ 3\ \bar{4}$	1.91	0.04	3.29	0.08	−4.5
25_{end}	14.2	$1\ 11\ \bar{2}$	2.00	0.02	3.14	0.04	

References

[1] V. R. Dubach and A. Guskov, *Crystals*, 2020, **10**, 580.
